# Supplementary material for: A retrospective cohort study of Paxlovid efficacy depending on treatment time in hospitalized COVID-19 patients
Source: eLife. 2024 Apr 16;13:e89801. doi: 10.7554/eLife.89801 (PMC11078542; doi:10.7554/eLife.89801)
Supplement: Supplementary file 1. — We used a nonlinear mixed-effects method to fit a within-host model of viral kinetics to the viral titer measurements from 208 patients, 104 were treated with Paxlovid and 104 did not receive any antiviral drugs (Traynard et al., 2020). The reported medians and variation across individuals (95% interpercentile ranges) integrate both fixed and random effects estimates for each parameter. [file elife-89801-supp1.docx]

**Supplementary File 1. Within-host model parameter estimates.** We used a nonlinear mixed-effects method to fit a within-host model of viral kinetics to the viral titer measurements from 208 patients, 104 were treated with Paxlovid and 104 did not receive any antiviral drugs [(Traynard et al., 2020)](https://paperpile.com/c/R88YH4/MRaJj). The reported medians and variation across individuals (95% interpercentile ranges) integrate both fixed and random effects estimates for each parameter.

| **Parameter** | **Estimated median [95% interpercentile range] across infected individuals** |
| --- | --- |
| Cell infection rate in 10^-9^ mL/Copies in days^-1^ ($\beta$) | 17.14 [12.60, 21.75] |
| Rate in log10 for the interferon-induced conversion of target cells to refractory cells (Φ) | -9.78 [-10.68, -5.54] |
| Rate in 10^-3^ at which refractory cells become target cells again (𝜌) | 5.27 [4.63, 6.00] |
| Infected cell clearance rate in days^-1^ ($\delta$) | 0.48 [0.28, 1.02] |
| Virus production rate in Copies/ mL in days^-1^ (𝜋) | 69.39 [55.75, 78.63] |
| Maximum antiviral efficacy ($\epsilon_{max}$) | 0.91 [0.91, 0.92] |
