# Supplementary material for: A retrospective cohort study of Paxlovid efficacy depending on treatment time in hospitalized COVID-19 patients
Source: eLife. 2024 Apr 16;13:e89801. doi: 10.7554/eLife.89801 (PMC11078542; doi:10.7554/eLife.89801)
Supplement: Supplementary file 2. — The table provides population-wide fixed and random effects estimates for the viral dynamic parameters, whereas Supplementary file 1 provides the estimated median and variation across individuals for each parameter. The values assume that antiviral efficacy follows a logit-normal distribution and all other parameters follow log normal distributions*. Values in parentheses are relative standard errors (RSE) as a percent. [file elife-89801-supp2.docx]

##### **Supplementary File 2. Population-wide parameter estimates for the within-host model.** The table provides population-wide fixed and random effects estimates for the viral dynamic parameters, whereas Supplementary File 1 provides the estimated median and variation across individuals for each parameter. The values assume that antiviral efficacy follows a logit-normal distribution and all other parameters follow log normal distributions*. Values in parentheses are relative standard errors (RSE) as a percent.

| **Parameter** | **Fixed effect** | **Random effect SD** |
| --- | --- | --- |
| Cell infection rate in 10^-9^ mL/Copies in days^-1^ ($\beta$) | 17.26 (12) | 0.49 (29) |
| Rate in log10 for the interferon-induced conversion of target cells to refractory cells (Φ) | -9.37 (110) | 0.81 (17) |
| Rate in 10^-3^ at which refractory cells become target cells again (𝜌) | 5.24 (28) | 0.46 (86) |
| Infected cell clearance rate in days^-1^ ($\delta$) | 0.49 (5) | 0.61 (7) |
| Virus production rate in Copies/ mL in days^-1^ (𝜋) | 68.58 (10) | 0.38 (26) |
| Maximum antiviral efficacy ($\epsilon_{max}$) | 0.91 (1) | 0.32 (43) |

* The estimation model is of the form $Y_{i}(t)={log}_{10}(V_{i}(t,\psi_{i}))+e_{i}(t)$, where $Y_{i}(t)$ is the measured viral load of patient $i$ at time $t$, $V_{i}(t,\psi_{i})$ is the actual viral load given viral dynamic parameters $\psi_{i}$ for patient $i$, and $e_{i}(t)$ is the residual term. For parameters $\psi_{i}$ following a log-normal distribution or logit-normal distribution, $\psi_{i}=\gamma\times e^{\eta_{i}}$ or ${logit(\psi}_{i})=logit(\gamma)+\eta_{i}$, respectively, where $\gamma$ are fixed effects and $\eta_{i}$ are random effects.
