# Supplementary material for: A retrospective cohort study of Paxlovid efficacy depending on treatment time in hospitalized COVID-19 patients
Source: eLife. 2024 Apr 16;13:e89801. doi: 10.7554/eLife.89801 (PMC11078542; doi:10.7554/eLife.89801)
Supplement: Supplementary file 3. — To estimate the probability of a rebound, we computed the fraction out of 1000 simulations in which the viral titer rebounded to higher values following treatment than observed prior to treatment. To estimate reduction in infectiousness, we run 1000 pairs of treatment versus no treatment simulations. Following each simulation, we calculate the total infectiousness from the time of infection until 15 DPOS using estimates relating viral titer to infectiousness provided in Marc et al., 2021. For each pair, we then compute the percent difference in total infectiousness of the treatment simulation relative to the no treatment simulation. The table reports medians and 95% CIs across all treated patients, patients who do not experience a post-treatment rebound, and patients who do. [file elife-89801-supp3.docx]

**Supplementary File 3. Estimated reduction in overall infectiousness and likelihood of a post-treatment rebound, depending on the timing of Paxlovid initiation in days post onset of symptoms (DPOS).** To estimate the probability of a rebound, we computed the fraction out of 1000 simulations in which the viral titer rebounded to higher values following treatment than observed prior to treatment. To estimate reduction in infectiousness, we run 1000 pairs of treatment versus no treatment simulations. Following each simulation, we calculate the total infectiousness from the time of infection until **15** DPOS using estimates relating viral titer to infectiousness provided in ref. [(Marc et al., 2021)](https://paperpile.com/c/R88YH4/mjIeK). For each pair, we then compute the percent difference in total infectiousness of the treatment simulation relative to the no treatment simulation. The table reports medians and 95% CIs across all treated patients, patients who do not experience a post-treatment rebound, and patients who do.

| **Treatment initiation time (DPOS)** | **Estimated probability of rebound** | **Estimated reduction in infectiousness due to Paxlovid (percent)** | | |
| --- | --- | --- | --- | --- |
|  |  | **All patients** | **Non-rebound patients** | **Rebound patients** |
| **-1** | 1.00 | 1 [0, 11] | 12 [9, 13] | 1 [0, 11] |
| **0** | 0.96 | 1 [-2, 15] | 12 [-1, 21] | 1 [-2, 13] |
| **1** | 0.74 | 3 [-4, 17] | 12 [-4, 20] | 1 [-4, 13] |
| **2** | 0.41 | 9 [-5, 17] | 12 [-4, 18] | 1 [-6, 14] |
| **3** | 0.17 | 11 [-3, 16] | 12 [0, 16] | 3 [-5, 14] |
| **4** | 0.05 | 11 [0, 15] | 11 [0, 15] | 5 [-2, 17] |
| **5** | 0.01 | 10 [0, 14] | 10 [0, 14] | 9 [4, 16] |
| 6 | 0 | 7 [0, 13] | 7 [0, 13] | NA |
| 7 | 0 | 5 [0, 12] | 5 [0, 12] | NA |
| 8 | 0 | 3 [0, 11] | 3 [0, 11] | NA |
| 9 | 0 | 1 [0, 10] | 1 [0, 10] | NA |
| 10 | 0 | 0 [0, 9] | 0 [0, 9] | NA |
