# Supplementary material for: A retrospective cohort study of Paxlovid efficacy depending on treatment time in hospitalized COVID-19 patients
Source: eLife. 2024 Apr 16;13:e89801. doi: 10.7554/eLife.89801 (PMC11078542; doi:10.7554/eLife.89801)
Supplement: Supplementary file 4. — Day zero corresponds to the first day of symptoms. [file elife-89801-supp4.docx]

**Supplementary File 4. The distribution of treatment initiation times for the 104 patients who received Paxlovid.** Day zero corresponds to the first day of symptoms.

| **Treatment initiation time (DPOS)** | **Percent of patients** |
| --- | --- |
| -1 | 0.96 |
| 0 | 0.00 |
| 1 | 0.00 |
| 2 | 0.96 |
| 3 | 5.77 |
| 4 | 25.96 |
| 5 | 29.81 |
| 6 | 17.31 |
| 7 | 8.65 |
| 8 | 7.69 |
| 9 | 0.00 |
| 10 | 2.88 |
